# Lipocalin-2 promotes breast cancer brain metastasis by enhancing tumor invasion and modulating brain microenvironment

**DOI:** 10.3389/fonc.2024.1448089

**Published:** 2024-08-12

**Authors:** Yang Zhao, Xiaogen Tang, Tingting Lei, Dongwei Fu, Hongyi Zhang

**Affiliations:** ^1^ Department of Microbiology and Immunology, School of Medicine, Jinan University, Guangzhou, Guangdong, China; ^2^ Department of Oncology, The Affiliated Shunde Hospital of Jinan University, Foshan, Guangdong, China

**Keywords:** lipocalin-2, breast cancer brain metastasis, blood-brain barrier, brain microenvironment, extracellular matrix (ECM)

## Abstract

Breast cancer is the leading cancer diagnosed in women globally, with brain metastasis emerging as a major cause of death, particularly in human epidermal growth factor receptor 2 positive and triple-negative breast cancer subtypes. Comprehensive understanding of the molecular foundations of central nervous system metastases is imperative for the evolution of efficacious treatment strategies. Lipocalin-2 (LCN2), a secreted iron transport protein with multiple functions, has been linked to the progression of breast cancer brain metastasis (BCBM). In primary tumors, LCN2 promotes the proliferation and angiogenesis of breast cancer cells, triggers the epithelial-mesenchymal transition, interacts with matrix metalloproteinase-9, thereby facilitating the reorganization of the extracellular matrix and enhancing cancer cell invasion and migration. In brain microenvironment, LCN2 undermines the blood-brain barrier and facilitates tumor seeding in the brain by modulating the behavior of key cellular components. In summary, this review meticulously examines the fuel role of LCN2 in BCBM cascade, and investigates the potential mechanisms involved. It highlights the potential of LCN2 as both a therapeutic target and biomarker, indicating that interventions targeting LCN2 may offer improved outcomes for patients afflicted with BCBM.

## 1 Introduction

Breast cancer (BC) is the most prevalent cancer in the women worldwide and ranks as the second highest cause of cancer-related mortality (1). Brain metastasis (BrM) stands out as a significant contributor to mortality in breast cancer patients, particularly in certain subtypes. An estimated 30–50% of patients with metastatic HER2-positive breast cancer and 25–46% of those with metastatic triple-negative breast cancer (TNBC) develop BrM (2–4). The current clinical approach for treating breast cancer brain metastasis (BCBM) involves a multidisciplinary strategy that encompasses surgery, stereotactic radiation therapy, and chemotherapy (5). Despite advancements in surgery and radiotherapy that have led to improved survival rates for patients with metastatic brain tumors, cognitive impairment and a decline in quality of life remain unavoidable challenges (6). Consequently, more effective systemic treatments are urgently required to enhance the management of BCBM.

The development of brain metastasis from cancer typically involves a series of well-defined stages: primary tumor growth and invasion, intravasation into lymph and blood vessels, survival of circulating tumor cells (CTCs) in the circulation, extravasation across blood-brain barrier (BBB), and ultimately colonization and proliferation within the brain (7–9). Targeted therapies such as tucatinib, neratinib, and pyrotinib have shown effective antitumor activity against metastatic brain tumors (10). However, BBB often prevents drugs penetrating the brain, limiting the efficacy of most treatments for brain metastases (11). Central nervous system (CNS) metastases have distinct molecular characteristics that differ from primary tumors and other metastases, reflecting tumor heterogeneity (12). Genetic variations in intracranial lesions can quickly lead to resistance to systemic therapies (13). Therefore, the thorough understanding of the molecular mechanisms underlying CNS metastases is essential for developing more effective treatments.

LCN2, also known as neutrophil gelatinase-associated lipocalin (NGAL), has been found to be systemically upregulated in the brain tissue of BCBM patients and is strongly correlated with disease progression and poor survival (14). It is a multifunctional protein belonging to the lipocalin superfamily, playing roles in the innate immune response, inflammatory response, iron homeostasis, lipid metabolism, tumor migration, and apoptotic signaling (15–17). LCN2 was initially identified as a 25-kDa human neutrophil protein isolated from the 135-kDa form of gelatinase, consisting of a 198-AA protein with a 20-AA signal peptide at the N-terminal and a 178-AA protein with glycosylation sites (18, 19). Its conserved three-dimensional (3D) structure, characterized by an 8-stranded β-barrel, allows LCN2 to bind to various ligands and receptors including megalin/GP330 in humans and SLC22A17/24p3R in mice, forming molecular complexes through disulfide bonds with neutrophil gelatinase B (18–22).

**Figure 1:**
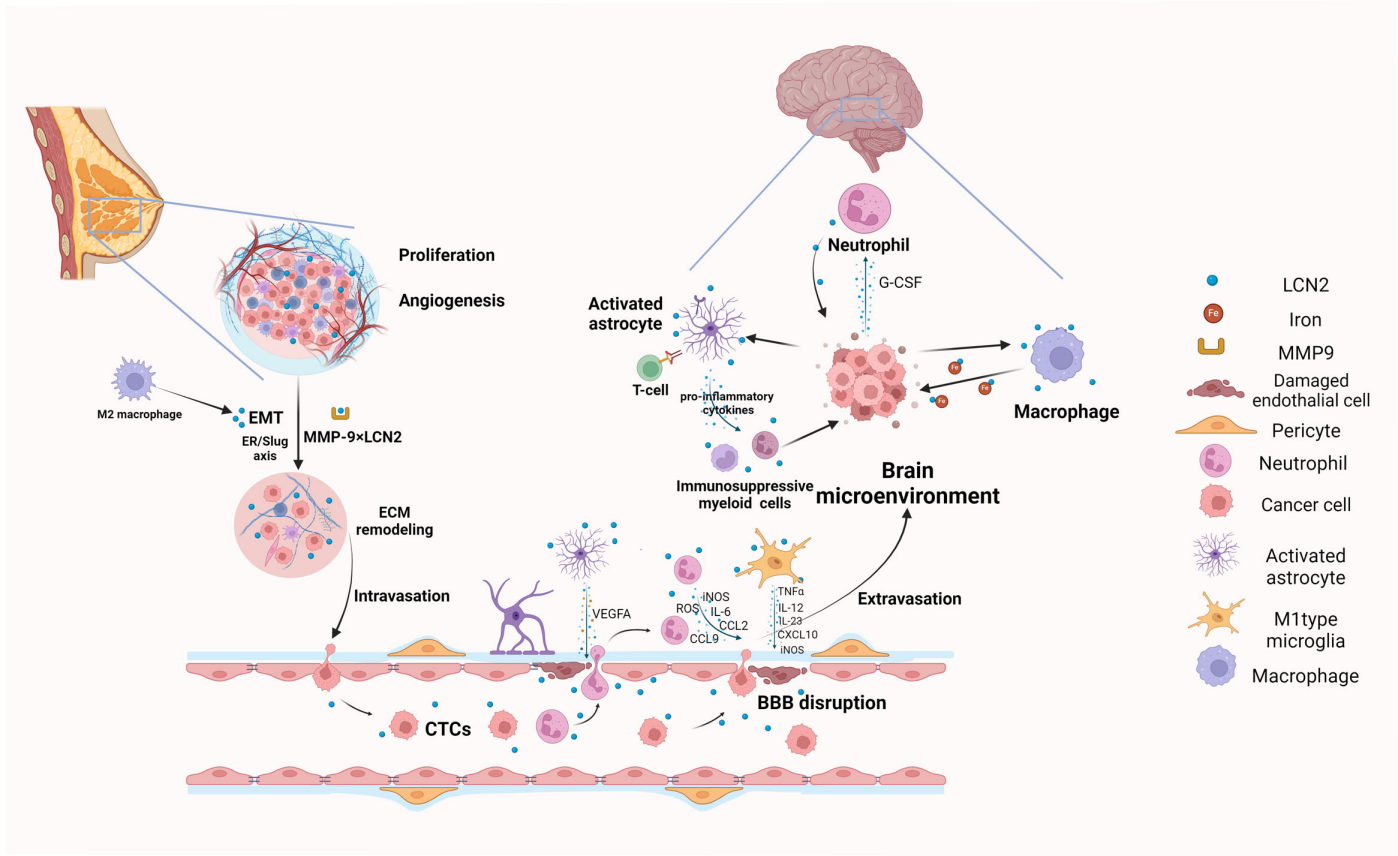
Graphical summary of biological mechanisms of LCN2 involved in breast cancer brain metastasis (Created with BioRender.com). LCN2 promotes local tumor growth and invasion through angiogenesis, EMT, and ECM remodeling, which are crucial preconditions for cancer cells to intravasate and migrate to the brain. During extravasation, LCN2 disrupts the BBB and facilitates CTCs in penetrating blood vessels into the brain by inducing the release of pro-inflammatory cytokines from astrocytes, microglia and neutrophils. Finally, the surviving cancer cells colonize and proliferate in the brain by modulating neuroinflammation and creating an immunosuppressive brain microenvironment. EMT, epithelial to mesenchymal transition; ECM, extracellular matrix remodeling; CTCs, Circulating tumor cells; BBB, Blood-Brain Barrier.

During inflammation and immune responses, LCN2 serves as a pivotal acute phase protein that is markedly upregulated (23). It transports iron by forming complexes with siderophores, aiding circulation while blocking reactivity (24, 25). In addition,LCN2 has the ability to limit bacterial growth by sequestering iron chelators during immune responses and acts as an antioxidant, protecting against oxidative stress and maintaining hypoferremia (26–30). Notably, intracellular expression of LCN2 has been shown to promote the progression of various tumors, such as breast cancer, colorectal cancer, cholangiocarcinoma (31, 32). Although extensive research indicates that LCN2 significantly contributes to progression of breast cancer, its specific role in breast cancer brain metastasis is not yet fully understood. In this review, we outline the multifaceted role of LCN2 in promoting breast cancer brain metastasis, highlighting its contribution to BBB disruption and the brain microenvironment (**Figure 1**).

## 2 LCN2 promotes growth and invasion of primary breast cancer

**Figure 2:**
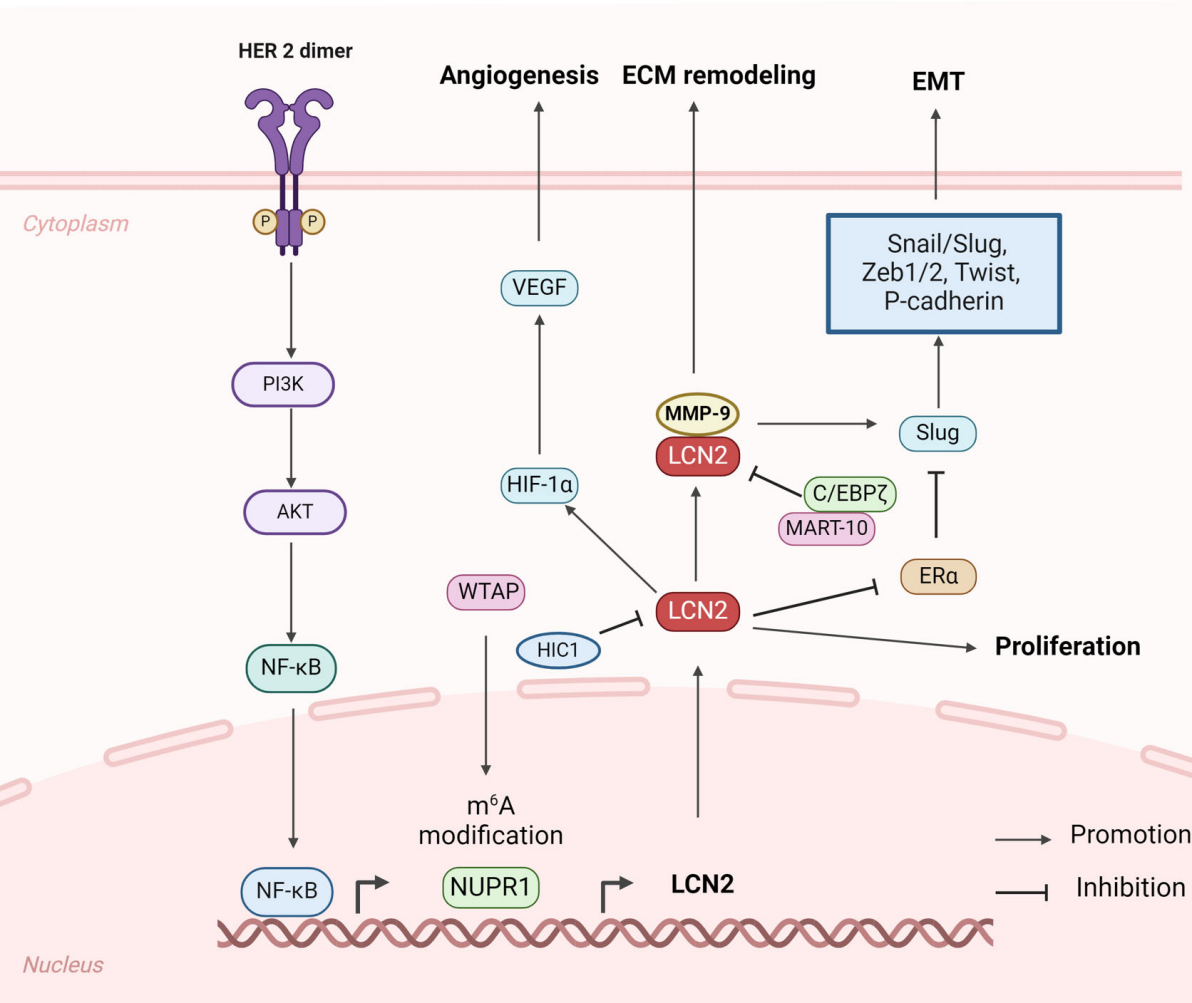
Structure and biomechanisms of LCN2 involved in primary breast cancer growth and invasion (Created with BioRender.com). In primary breast cancer, LCN2 emerges as a critical player influencing multiple aspects of tumor biology, including promoting tumor growth, angiogenesis, EMT, and ECM remodeling.

Primary tumor growth and invasion are crucial in the development of BCBM, as they provide the initial population of cancer cells that can disseminate to distant sites (33). Studies have shown that LCN2 is actively involved in these processes by promoting cellular proliferation, angiogenesis, EMT and ECM remodeling, thereby enhancing the metastatic potential of breast cancer cells (**Figure 2**) (34). Consequently, understanding the role of LCN2 in primary tumor growth and invasion is vital for developing targeted therapies to prevent BCBM.

### 2.1 Proliferation and angiogenesis

The heterogeneity of LCN2 expression was initially validated through meticulous immunohistochemical analysis of MCF-7 tumor samples, which revealed that tumors with elevated LCN2 levels exhibited accelerated growth rates, enhanced angiogenesis, and a significantly higher proportion of proliferating tumor cells (35). The overexpression of HER2 orchestrates the upregulation of LCN2 via the HER2/AKT/NF-κB signaling cascade, which is intimately associated with heightened tumorigenesis and progression in breast cancer patients (36, 37). In the context of inflammatory breast cancer (IBC), a reduction in LCN2 expression was found to markedly suppress tumor growth, invasion, and metastatic spread to the brain in both *in vitro and in vivo* studies (38). Furthermore, Wilms tumor 1-associated protein (WTAP) mediated Nuclear protein-1 (NUPR1) was identified as a positive regulator of LCN2 expression through m6A modification, fostering TNBC cellular proliferation, migration, and invasion by inhibiting ferroptosis (39). These findings underscore the significant upregulation of LCN2, thereby driving enhanced proliferation and progression across diverse breast cancer cell lines.

Angiogenesis and lymphangiogenesis are pivotal in BCBM, as they facilitate the formation of new blood and lymphatic vessels, providing essential pathways for cancer cell dissemination and establishment in the brain (40). To stimulate angiogenesis, vascular endothelial growth factor (VEGF), a crucial angiogenic activator, is indispensable for the angiogenic activity (41). Recent studies have found that LCN2 expression significantly elevates VEGF levels by inducing hypoxia-inducible factor 1 (HIF-1α) expression through the extracellular signal-regulated kinase (Erk) pathway in both MCF-7 human breast cancer cells and an angiogenic line derived from MDA-MB-436 cells (42). Furthermore, the Matrix metalloproteinase-9 (MMP-9)/LCN2 complex was also identified in MCF-7 cells, significantly contributing to enhanced cell proliferation and angiogenesis (35). During tumor lymphangiogenesis, tumor-associated macrophages (TAMs) release VEGF-C/D, which increases tumor lymphangiogenesis by interacting with their receptor VEGFR3 on lymphatic endothelial cells (LECs) (43, 44). Phospholipid sphingosine 1-phosphate (S1P), released from dying breast tumor cells, activates the STAT3 consensus sequence at the LCN2 promoter, promoting the expression of LCN2 in TAMs by S1P receptor 1 (S1PR1) (45). TAM-derived LCN2 has been shown to induce LEC proliferation and lymphangiogenesis, through the subsequent induction of the VEGFC-VEGFR3 interaction in LECs. Deletion of LCN2 significantly inhibited lymphatic vessel growth surrounding tumors and decreased metastasis of breast tumors in both MMTV-PyMT mice and mice with orthotopic wild-type tumors (45). These findings highlight the crucial role of LCN2 in regulating VEGF-mediated angiogenesis and promoting tumor lymphangiogenesis through the induction of VEGFC-VEGFR3 interaction, emphasizing its potential as a therapeutic target in breast cancer.

### 2.2 Epithelial-mesenchymal transition (EMT)

EMT is a biological process during which epithelial cells transform into motile mesenchymal cells, thereby acquiring enhanced capabilities for proliferation and migration (46). This transformation involves an increase in mesenchymal markers such as vimentin and fibronectin, coupled with a decrease in the epithelial marker E-cadherin (47, 48). The CRISPR-mediated knockout of LCN2 in human TNBC cells resulted in a marked reduction in TNBC aggressiveness, primarily by modulating the EMT process and inhibiting cell migration (49). In MCF-7 cells, the silencing of PTEN results in the upregulation of LCN2 and MMP-9, which subsequently leads to elevated expression of EMT-associated transcription factors, including Snail/Slug, Zeb1/2, Twist, and P-cadherin (48). Conversely, treatments with MART-10 and 1α,25(OH)2D3 suppressed the expression of LCN2 and MMP-9 and reversed the EMT process by reducing the expression of Zeb1, Zeb2, Snail, and Slug in MDA-MB-231 cells (50). Furthermore, estrogen receptor α (ERα) has been noted to negatively regulate Slug (47), and a negative correlation was observed between LCN2 expression and the expression of ERα and progesterone receptor (PR) in primary breast tumors (51, 52). Collectively, these findings suggest that LCN2 may induce EMT via the ER/Slug pathway, beginning with the downregulation of ERα, leading to the upregulation of Slug expression, and culminating in the acquisition of a mesenchymal phenotype (53).The role of LCN2 in EMT within the tumor microenvironment (TME) has also been investigated. In both PyMT-mouse models and human breast tumors, LCN2 expression predominantly occurs in the tumor stroma rather than within tumor cells (54). Tumor stroma-derived LCN2, particularly that secreted by M2 macrophages via STAT3 and C/EBPβ signaling pathways, induces EMT in MCF-7 breast cancer cells, thereby enhancing their migration and invasion capabilities in both *in vitro and in vivo* models (55). Within the TNBC microenvironment, LCN2 released by macrophages, fibroblasts, and endothelial cells significantly promotes the proliferation and migration of TNBC cells (56). These findings underscore the pivotal role of LCN2 in facilitating EMT, emphasizing the necessity for further investigation to fully elucidate the underlying mechanisms and potential therapeutic targets.

### 2.3 Extracellular matrix (ECM) remodeling

LCN2 interacts with MMP-9 to form a 125-kDa urinary MMP-9/LCN2 complex, which significantly contributes to the progression and metastasis of various tumors (57). During tumor progression, MMP-9 facilitates the invasion of tumor cells into the basement membrane by degrading specific substrates such as gelatin, elastin, and collagens, thereby promoting tumor metastasis and dissemination (58, 59). In the MMTV-PyMT model, activated MMP-9 has been shown to facilitate breast cancer cell migration, invasion, and metastasis to lung niche (60). In human breast cancer, the MMP-9 x LCN2 complex was first identified by antibody detection and has been found in the urine of breast cancer patients as opposed to healthy individuals (35, 61). Overexpression of LCN2 in MCF-7 and HER2-positive breast cancer cells is associated with increased levels of MMP-9, enhanced stabilization and activation of its enzymatic activity, and protection from degradation (34). This interaction enhances tumor proliferation, invasion, migration, and angiogenesis (35, 37, 61). Prior research indicates that the downregulation of LCN2 and MMP-9 can significantly suppress breast cancer migration and invasion. This suppression can be achieved by the overexpression of the transcriptional factor CCAAT/enhancer-binding protein ζ (C/EBPζ), as well as treatment with 1α,25(OH)2D3 and its newly synthesized analog MART-10 (50, 62). Clinical studies have further corroborated this interaction, suggesting that LCN2 could serve as a predictive biomarker for MMP-9 levels and the progression of breast cancer (35, 63). Furthermore, LCN2 is identified as a direct target gene of Hypermethylated in Cancer 1 (HIC1), a tumor suppressor specifically active in TNBC. LCN2 can partially rescue HIC1-induced reductions in cell invasion and metastasis with no effect on the regulation of EMT or MMP9 activity, which suggests that the function and potential mechanisms of LCN2 in breast cancer need to be further elucidated (64).

In primary breast cancer, LCN2 significantly contributes to the growth by promoting proliferation, lymphangiogenesis and angiogenesis. It also induces breast cancer cells to undergo EMT. Furthermore, LCN2 can interact with MMP-9 to facilitate the degradation of extracellular substrates. This transformation, coupled with the MMP-9 x LCN2 complex, enables the cells to remodel their surrounding ECM and enhance tumor cell invasion. These capabilities equip the cells to infiltrate adjacent tissues and subsequently penetrate the blood vessel endothelium to become circulating tumor cells (CTCs), either as individual cells or in clusters.

## 3 LCN2-mediated blood-brain barrier disruption

CTCs are released from primary tumors and travel through the bloodstream or lymphatic system before lodging in distant tissues (65). To successfully colonize and proliferate within the brain, CTCs must traverse the BBB (66, 67). The BBB is a complex, dynamic, and selectively permeable structure that lines the blood vessels in the brain. It serves as a physical and metabolic barrier between the bloodstream and the neuroglia of the CNS parenchyma (68). The structural integrity of the BBB is maintained by a complex elements including tightly connected ECs, astrocytic endfeet, pericytes, and various ECM components (69). These elements interact to regulate permeability, provide structural support, and ensure selective barrier function, protecting the CNS from harmful substances (70).LCN2 is upregulated in key cellular components of the BBB such as cerebral endothelial cells, neutrophils, and astrocytes, while its receptor 24p3R is expressed in oligodendrocytes, astrocytes, endothelial cells, and pericytes, particularly in brain metastasis (71, 72). Previous research indicates that LCN2 increases BBB permeability by significantly raising capacitance and reducing transendothelial electrical resistance (TEER), thus enhancing BBB leakage (73). However, the potential mechanisms by which LCN2 disrupts the BBB are still unclear.

A major factor contributing to BBB disruption is uncontrolled inflammation following injuries or diseases. Recent work has shown that blocking pro-inflammatory cytokines, including TNFα, IL-1β, and IL-6, can reduce the permeability of BBB (74). Elevated levels of circulating LCN2 stimulate the release of pro-inflammatory cytokines IL-6 and IL-1β in brain endothelial cells, leading to BBB disruption by reducing the expression of tight junction proteins such as claudin-5 and ZO-1 (75). Astrocytes play a decisive role in maintaining and regulating the integrity of the BBB by directly interacting with endothelial cells and modulating the barrier function through their foot processes and organic anion transporters (76). Recent work has shown that LCN2 activate astrocytes and induce the expression of VEGFA, thereby influencing BBB permeability via the activation of the downstream effector eNOS (77, 78). Another pathway is astrocyte-derived VEGFA, which can increase the BBB permeability by down-regulating the endothelial transmembrane tight junction proteins claudin-5 and occludin, resulting in increased paracellular permeability and loss of barrier function (79). In other components, recent studies indicate that LCN2 contributes to BBB disruption by promoting neutrophil infiltration, which exacerbates BBB disruption through the release of reactive oxygen species (ROS) and proteases (80). Neutrophils release ROS and facilitate other cells to produce cytokines, attracting more leukocytes from the periphery. The recruitment of inflammatory cells is aggravated by ROS-induced NF-κB-mediated upregulation of adhesion molecules, propagating an inflammation cascade that further promotes BBB disruption (81, 82). Neutralizing LCN2 with a monoclonal antibody has been shown to reduce the production of pro-inflammatory mediators such as iNOS, IL-6, CCL2, and CCL9, as well as neutrophil infiltration, resulting in reduced BBB leakage (71). After all, the underlying intercellular signaling pathways, as well as strategies for using these effects to keep the integrity of BBB are promising targets for future study.

**Figure 3:**
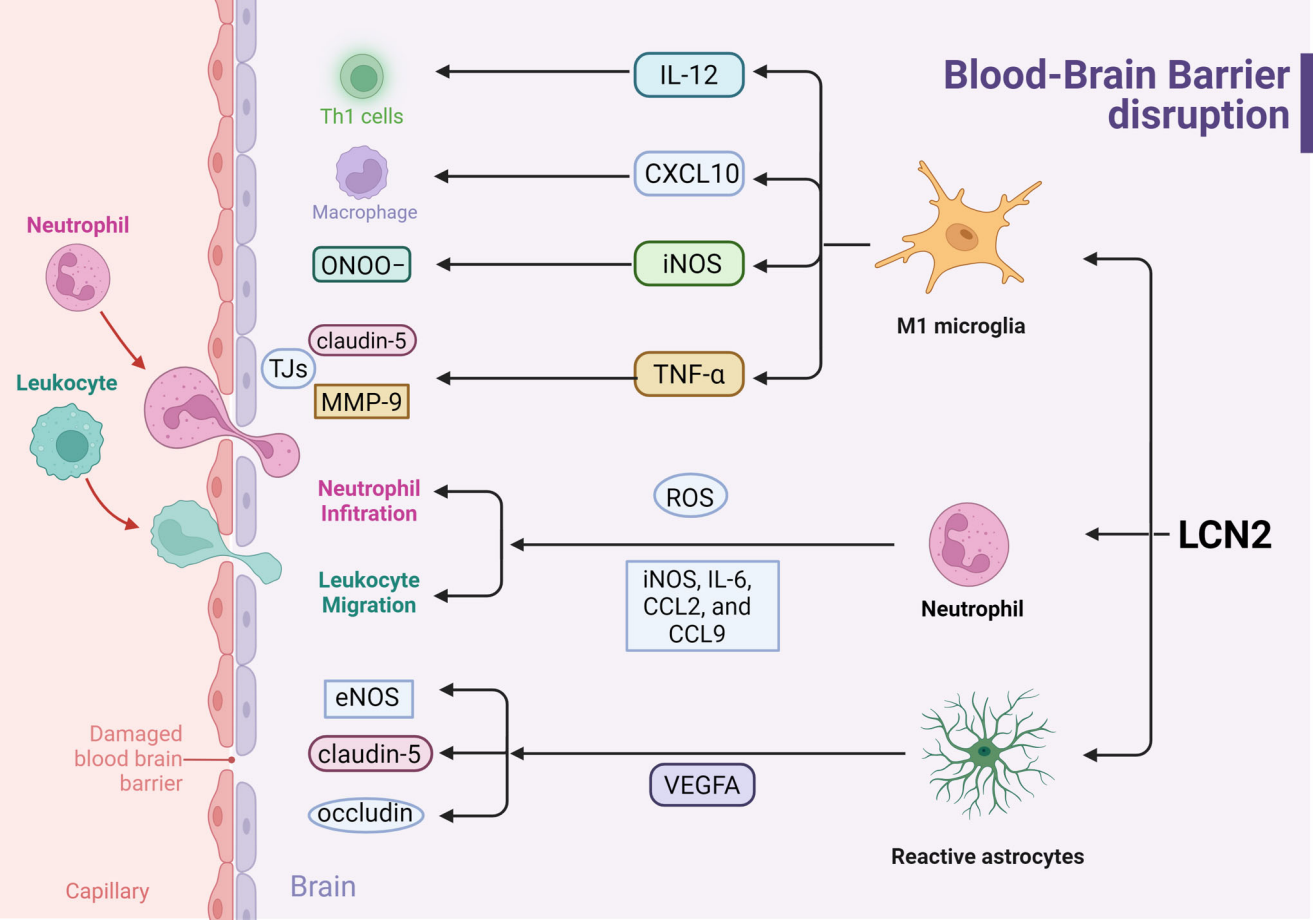
Effects of LCN2 on Blood-Brain Barrier disruption during inflammation response (Created with BioRender.com). LCN2 disrupts the BBB by modulating the activation of astrocytes, microglia, and neutrophils, thereby amplifying the pro-inflammatory response and releasing inflammatory factors, ultimately leading to BBB leakage.

Microglia, the long-lived resident immune cells of the brain, are crucial for BBB function. M1 microglia exacerbate BBB disruption, whereas M2 microglia aid in repairing BBB damage (83). Under this condition,LCN2 amplifies pro-inflammatory M1 polarization of activated microglia, with increased the M1-related gene expression including IL-12, IL-23, iNOS, TNF-α, and CXCL10 in cultured mouse microglial cells without affecting M2-markers such as IL-10, Arg1, and Mrc1 (84). IL-12 and TNF-α, secreted by M1 type microglia, accelerate the polarization of Th1 cells and increase BBB permeability (80). In M1 microglia, secreted chemokines CXCL10 promote BBB disruption and acts as a chemoattractant protein that facilitates monocyte and macrophage migration across the BBB (85). Another way, iNOS, upregulated by M1 microglia, lead to the production of nitric oxide (NO), which can combine with superoxide anion (O2−) to form peroxynitrite (ONOO−). Peroxynitrite causes extensive damage to neurons and cerebral microvessels, contributing to BBB disruption through lipid peroxidation, depletion of antioxidants, DNA fragmentation, and mitochondrial failure (86). Furthermore, recent research indicates that microglia-derived TNF-α interacts with TNFR-1 to disrupt tight junctions (TJs) and induce BMEC necroptosis (87). The ability of TNF-α to increase BBB permeability after CNS injuries and metastasis is well established. TNF-α disrupts BBB in two ways: First, TNF-α reduces claudin-5 levels via the NF-κB signaling pathway and disrupts TJs through the activation of p38MAPK and MEK1/2-ERK1/2 pathways (88, 89). Second, TNF-α also upregulates MMP-9 via the Ca/CAMK II/ERK/NF-κB signaling pathway, degrading TJs and the basal membrane of the BBB, further compromising BBB integrity (90). In short, Microglia-derived LCN2 amplifies pro-inflammatory M1 polarization, exacerbating BBB disruption through multiple mechanisms, including cytokine secretion, chemokine promotion, and oxidative damage, ultimately compromising BBB integrity. In summary, LCN2 disrupts the BBB by activating astrocytes, polarizing microglia, and promoting neutrophil infiltration, making it a crucial target for preserving BBB integrity in brain metastasis (**Figure 3**).

## 4 LCN2 facilitates tumor seeding in the brain microenvironment

The brain is composed of two main microenvironments: the densely cellular parenchyma and the cerebrospinal fluid (CSF)-filled leptomeninges (91).The brain parenchyma hosts cell types unique to the CNS, including astrocytes, microglia, oligodendrocytes, and neurons, which together form a complex network (91). Thus, interactions between cancer cells and these brain-specific cell types are unique to brain metastases and stromal gene expression changes significantly between normal and metastatic brain tissue (92). Analyzing the composition of BrM niches reveals these immunosuppressive states with enriched infiltrated T cells and macrophages in BCBM (93). These immunosuppressive cells such as FOXP3+ regulatory T cells, LAMP3+ tolerogenic dendritic cells, CCL18+ M2-like macrophages, RGS5+ cancer-associated fibroblasts, and LGALS1+ microglial cells are significantly reprogrammed, with interactions of immune checkpoint molecules LAG3-LGALS3 and TIGIT-NECTIN2 between CD8+ T cells and cancer/immune/stromal cells that play dominant roles in immune escape (94). Additionally, 3D organoids show that cancer-associated fibroblasts (CAF) in human BrM attract breast cancer cells via chemokines CXCL12 and CXCL16 (95). Within the TME of BCBM, CAF exhibit high expression of type I collagen genes and dominate cell-cell interactions via the type I collagen signaling axis, facilitating the remodeling of the TME to a collagen-I-rich ECM (94). Furthermore, GFAP+ reactive astrocytes expressing phosphorylated STAT3 (pSTAT3+ GFAP+ cells) are essential for BrM colonization and outgrowth via Chi3L1, a STAT3 target gene expressed by stromal cells in the BrM microenvironment (96).Taken together, the unique interactions between cancer cells and brain-specific stroma cell types, along with the immunosuppressive microenvironment in brain metastases, play crucial roles in immunosuppression and tumor progression.

**Figure 4:**
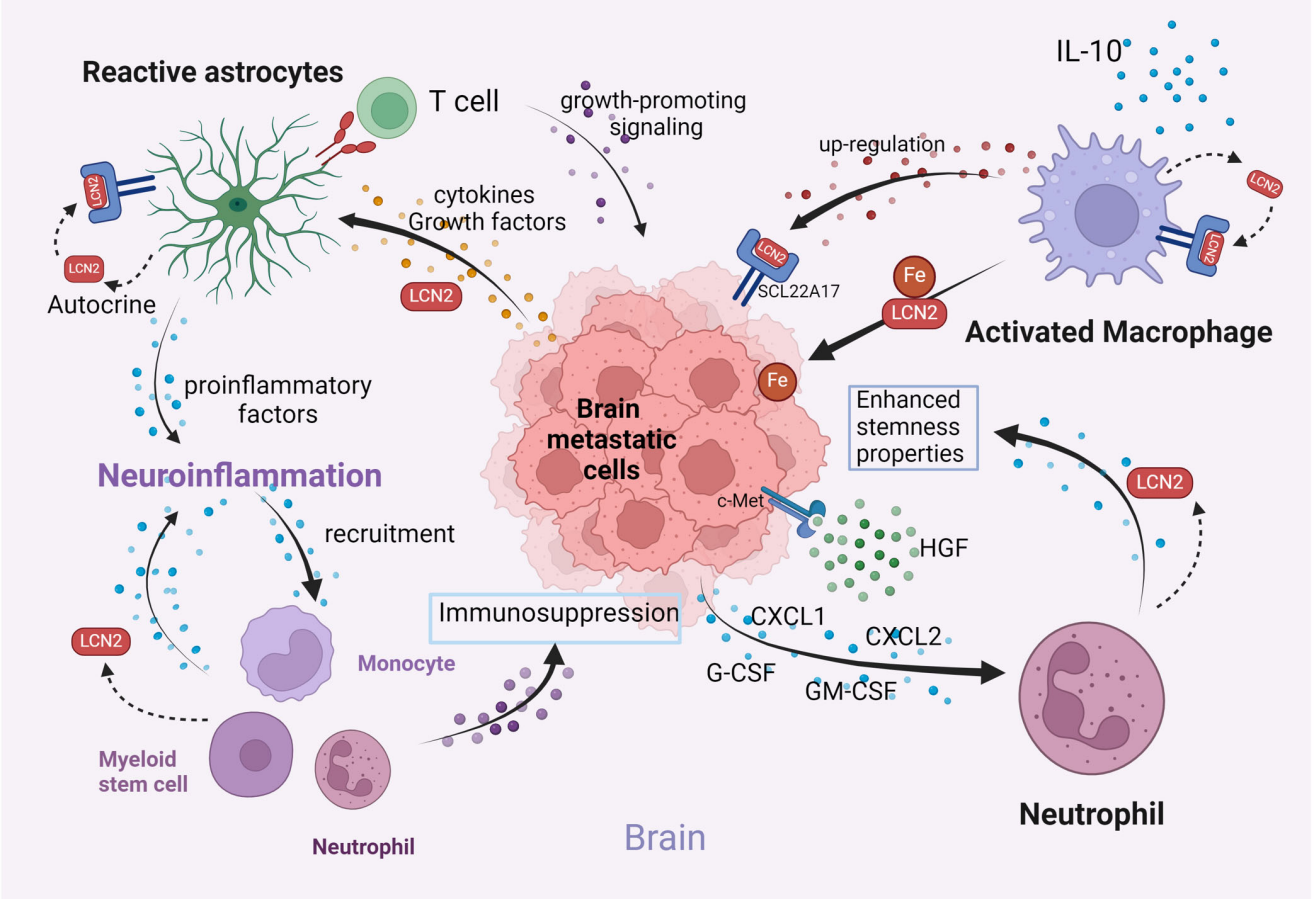
Illustration of the interactions among key components in the brain microenvironment (Created with BioRender.com). LCN2 facilitates tumor seeding in the brain microenvironment by activating astrocytes, promoting neuroinflammation, enhancing iron availability via macrophage secretion, and promoting neutrophil recruitment. These mechanisms collectively promote brain metastasis progression.

Interactions between microglia and metastatic cancer cells, astrocytes, and other immune cells in the brain parenchyma are involved in multiple processes associated with brain metastasis, including inflammation, angiogenesis, and immune modulation (97). In brain parenchyma, LCN2 is predominantly expressed and secreted by reactive astrocytes and activated microglia through autocrine and paracrine mechanisms. Inflammatory stimulation can increase the expression and secretion of LCN2, which then acts in an autocrine manner to induce morphological changes and sensitization processes in astrocytes and microglia (98, 99). Under disease conditions, LCN2 has been demonstrated to activate both astrocytes and microglia and modulate their production of both anti- and pro-inflammatory cytokines by regulating iron accumulation, mediating the regulation of neuroinflammation and neurotoxicity (17). The interactions of LCN2 in the brain microenvironment of BrM, specifically its impact on astrocytes, as well as the recruitment of macrophages and neutrophils, are investigated in this context (**Figure 4**).

### 4.1 Astrocytes

In the CNS, astrocytes are the primary cellular source for LCN2 expression (100). Studies have shown that LCN2 expression in astrocytes is induced by proinflammatory stimuli rather than anti-inflammatory stimuli (101). The LCN2 derived from classically activated astrocytes promotes further proinflammatory activation (102). These results support the notion that LCN2 promotes the classical activation of astrocytes in an autocrine manner (103). LCN2-activated astrocytes in brain metastasis are induced by various cytokines and growth factors secreted by cancer cells. Once recruited and activated by brain metastasizing cells, reactive astrocytes induce growth-promoting signaling in tumor cells by expressing PD-L1 to inhibit T-cell activation and secrete inflammatory factors, including lipocalin-2, directly suppressing immune cells (104). LCN2 also acts as a signaling molecule that transmits signals from the periphery to the brain, promoting neuroinflammation through astrocyte activation during breast cancer brain metastasis (105). Increased circulatory LCN2 upregulates the expression of the LCN2 receptor (24p3R) on astrocytes, microglia, brain endothelial cells, and the secretion of damage associated molecular pattern protein (DAMP) and high mobility group box 1(HMGB1), which subsequently induces oxidative stress and nod-like receptor protein 3 (NLRP3) inflammasome activation (75). Furthermore, elevated systemic levels of LCN2 in blood can initiate neuroinflammation by activating astrocytes, promoting brain metastasis by recruiting immunosuppressive myeloid monocytes and granulocytes, which in turn secrete LCN2, exacerbating neuroinflammation in brain metastasis (14). Collectively, these results indicate that elevated LCN2 stimulates the activation of astrocytes, leading to neuroinflammation and an enhanced release of pro-inflammatory cytokines and chemokines, which can be exploited by tumor cells to promote metastases formation and growth in brain metastasis.

### 4.2 Microglia/macrophage

Upon interacting with tumor cells, TAMs develop an iron-releasing phenotype, thereby augmenting the iron supply in the TME (106). The expression of iron-bound LCN2 in stromal cells and macrophages is associated with the initiation of tumors, metastases, and recurrence, independently of ferroportin (107). In both PyMT-mouse tumors and primary human breast tumors, LCN2 is primarily expressed in the tumor stroma rather than in the tumor cells themselves. Macrophage-derived LCN2 is crucial for iron transport into the TME, significantly enhancing tumor cell proliferation (108). Studies show that iron bound to LCN2 released by macrophages increases MCF-7 tumor cell proliferation, while LCN2 knockdown reduces both iron release and cell proliferation (109). Additionally, LCN2 depletion reduces FPN expression, suggesting a cooperative role in regulating iron export from TAM. These findings highlight the pivotal role of LCN2 in tumor iron metabolism and progression (110). Moreover, IL-10 has been demonstrated to polarize macrophages toward a regulatory M2 phenotype, upregulating the protumorigenic protein LCN2 through a STAT3 and C/EBPβ-dependent mechanism (111). However, in breast cancer leptomeningeal metastasis, macrophages do not produce LCN2 themselves. Instead, they induce cancer cells within the CSF to upregulate the expression of LCN2 and its receptor SCL22A17. This upregulation enables the collection of limited extracellular iron, thereby promoting breast cancer cell growth in the hypoxic leptomeninges (112).

### 4.3 Neutrophils

The intricate interplay between innate immune cells and tumor progression within the brain remains elusive. Notably, in brain metastatic sites, the increased presence of neutrophils significantly correlates with the expression of c-Met, which can be activated by its cognate ligand—hepatocyte growth factor (HGF) in tumor cells (113).The elevated activation of the c-Met pathway in brain metastatic cells leads to the upregulation of inflammatory cytokines like G-CSF, GM-CSF, CXCL1, and CXCL2. Consequently, these cytokines enhance the infiltration and survival of neutrophils within the brain microenvironment. Furthermore, c-Met-mediated cytokine signaling reprograms neutrophils into the N2 phenotype and modulates LCN2 expression in neutrophils, significantly enhancing the stemness properties of brain metastatic cell lines. Among these cytokines, tumor cell-derived G-CSF stands out as the primary cytokine responsible for upregulating LCN2 expression in neutrophils and inducing their reprogramming into immunosuppressive traits (114). Furthermore, LCN2 released by N2-neutrophils also promotes the mesenchymal-epithelial transition (MET) of breast cancer cells during metastasis via ERK/KLF4 signaling, thereby facilitating colonization and metastatic outgrowth (52). Collectively, the recruitment and modulation of neutrophils by c-Met high brain metastatic cells in metastatic sites promote brain metastasis through G-CSF-mediated LCN2 secretion in theTME.

## 5 Interactions between LCN2 and TME in preclinical models

Recent advancements in preclinical models have significantly enhanced the ability to study and treat heterogeneous tumors by accurately replicating the diverse TME in human cancer models (115, 116).Immune cells from blood or patient tumors have been reconstructed with heterogenic established cancer cell lines in conventional monolayer, spherical, or primary organoid cultures. However, these traditional vitro tumor models do not fully retain the diversity and physical structure of the complex TME, particularly lacking the capability to co-culture primary tumor epithelium with their natural infiltrating immune population (117). In recent years, the development of various 3D models, including 3D spheroid models, organoids, and organ-on-chip systems, has improved the study of physiologically relevant TME interactions through integrating different systems and facilitated high-throughput screening platforms for anti-cancer drug discovery and development (118, 119). These advancements have revolutionized biomedical research by replicating the complex TME *in vitro*, enabling detailed studies of tumor biology, cell interactions, and disease progression through advanced microphysiological systems (120). LCN2 has been implicated in regulating TME interactions and tumor progression in well-defined preclinical models.

Using a 3D spheroid model in which MCF-7 spheroids were treated with either LCN2-deficient or LCN2-containing macrophage-conditioned medium and then embedded in a collagen I matrix, it was confirmed that macrophage-derived LCN2 induces EMT and enhances the migration and invasion of MCF-7 breast cancer cells into the ECM (55). Notably, macrophage-derived LCN2 was found to donate iron to cancer cells, enhancing tumor growth, in a 3D tumor spheroid model established by stable LCN2R knockdown MCF-7 and MDA-MB-231 breast cancer cells (107). In another study using human cerebral organoids and direct astrocyte cultures, LCN2 synthesis in human neural cells was found to depend on the presence of microglia, with conditioned media from LPS-stimulated microglia significantly increasing LCN2 levels (121). In glioblastoma models, co-culturing glioblastoma cells with microglia resulted in significantly higher LCN2 levels and increased nuclear NFκB and STAT3 phosphorylation compared to monoculture, suggesting that microglia stimulate glioblastoma through LCN2 modulation (121). Furthermore, cytokine array and RNA sequencing analysis identified the LCN2 as a key factor in remodeling TME in the hepatocellular carcinoma (HCC)-on-a-chip model. LCN2 targeted therapy demonstrated robust anti-tumor effects in both the *in vitro* 3D biomimetic chip and *in vivo* mouse model, including inhibition of angiogenesis, promotion of sorafenib sensitivity, and enhancement of nature killer (NK)-cell cytotoxicity (122). Taken together, these innovations in 3D culture technologies and preclinical models have provided powerful tools for understanding the intricate interactions of LCN2 within the TME. Although the role of these preclinical models in LCN2-mediated brain metastasis has not yet been clearly studied, they suggest that in various tumors, different cells within the microenvironment regulate LCN2 expression to promote tumor progression.

## 6 LCN2-mediated resistance to radiotherapy and chemotherapy

Chemotherapy and radiotherapy are among the most common therapeutic tools used in cancer treatment, typically following the surgical removal of tumors (123). However, several barriers have diminished the effectiveness of these treatments. One major issue leading to the failure of both chemo- and radiotherapy is the resistance of tumor cells to anti-cancer drugs and X-ray irradiation (124). LCN2 has been increasingly identified for its significant role in modulating the response to radiotherapy and chemotherapy across various cancer types (**Table 1**) (134).

**Table 1:** The influence of LCN2 on the response to radiotherapy and chemotherapy.

Author	Year	Therapy Type	Cancer Type	LCN2 Role	Mechanism
Shiiba et al. (125)	2013	Radiotherapy	Oral Squamous Cell Carcinoma	Increases radioresistance	Possibly involved in PI3K/Akt pathway
Shiiba et al. (125)	2013	Radiotherapy	Lung Cancer	Increases radioresistance	Needs further research
Zhang et al. (126)	2014	Radiotherapy	Nasopharyngeal Carcinoma	Promotes radioresistance	Likely interacts with HIF1A
Yu et al. (127)	2014	Chemotherapy	Renal Cell Carcinoma	Mediates resistance to sunitinib	Mediates resistance to tyrosine kinase inhibitor
Zheng et al. (128)	2009	Chemotherapy	Glioblastoma	Implicated in resistance to BCNU	Role in Akt dephosphorylation, crucial for apoptosis sensitization
Monisha et al. (129)	2018	Chemotherapy	Oral Squamous Cell Carcinoma	Increases resistance to cisplatin	Reduces drug-induced oxidative stress
Chaudhary et al. (32)	2021	Chemotherapy	Colorectal Cancer	Enhances chemoresistance to 5FU	Inhibits ferroptosis by lowering iron levels and increasing glutathione peroxidase 4 and xCT expression
Kim et al. (130)	2017	Chemotherapy	Breast Cancer	Induces 5FU resistance via Wnt signaling	Participates in the activation of the Wnt signaling pathway
Shi et al. (131)	2024	Chemotherapy	Non-Small Cell Lung Cancer	Mediates resistance to almonertinib	Involves in LCN2-MMP-9 signaling pathway
Jiang et al. (132)	2023	Chemotherapy	Endometrial Cancer	Facilitates cisplatin(DDP) resistance	Regulated by HNRNPA2B1-mediated modifications influencing ferroptosis
Zhang et al. (133)	2022	Chemotherapy	Pancreatic Cancer	Regulates chemosensitivity to gemcitabine	Needs further research

PI3K, Phosphoinositide 3-kinase; HIF1A, Hypoxia-inducible factor 1-alpha; Akt, Protein Kinase B (PKB); 5FU, 5-fluorouracil; xCT, cysteine glutamate antiporter; Wnt, Wingless/Integrated; MMP-9, Matrix metalloproteinase-9; HNRNPA2B1, N6-methyladenosine (m6A) “reader”.

Research highlights that the expression of LCN2 increases substantially following X-ray irradiation, suggesting its involvement in cellular stress responses (135). The expression of LCN2 in oral squamous cell carcinoma (OSCC) cells and lung cancers was significantly upregulated by X-ray irradiation, possibly involved in PI3K/Akt pathway. Further investigations using small interfering RNA to silence LCN2 in OSCC cells and lung cancer cells revealed an increase in radiosensitivity, indicating that LCN2 defends cells against extracellular stimuli and facilitates cell survival (125). In nasopharyngeal carcinoma (NPC), LCN2 was highly expressed in the radioresistant NPC cell line CNE2R. Reducing LCN2 levels enhanced the radiosensitivity of NPC cells by impairing their DNA repair and proliferation abilities. Conversely, ectopic expression of LCN2 further promoted radioresistance in NPC cells, likely through interactions with hypoxia-inducible factor 1-alpha (HIF1A) (126). Although LCN2 has been implicated in promoting radioresistance in several cancer types, further research is necessary to fully understand its role and mechanisms across a broader range of tumors.

LCN2 also appears pivotal in chemotherapy resistance. In renal cell carcinoma, LCN2 mediated resistance to the tyrosine kinase inhibitor sunitinib (127). Similarly, in glioblastoma, LCN2 is implicated in resistance to BCNU (carmustine), primarily through its role in Akt dephosphorylation, which is crucial for apoptosis sensitization (128). Additionally, silencing LCN2 in OSCC leads to increased resistance to cisplatin by reducing the drug-induced oxidative stress, an effect typically exacerbated by LCN2’s influence on intracellular iron levels (129). When LCN2 is over-expressed, it leads to resistance to 5-fluorouracil in colon cancer cell lines both *in vitro* and *in vivo* by inhibiting ferroptosis through decreasing intracellular iron levels and stimulating the expression of glutathione peroxidase 4 and a component of the cysteine glutamate antiporter, xCT (32). Enhanced expression of LCN2 and activation of the Wnt signaling pathway may induce 5−FU resistance in RNA polymerase II transcription elongation factor (Ell3) over−expressing MCF−7 cells, allowing them to evade apoptosis (130). Moreover, LCN2 has been linked to acquired resistance to almonertinib in non-small cell lung cancer (NSCLC) through the LCN2-MMP-9 signaling pathway and its regulation by HNRNPA2B1-mediated mA modification of FOXM1 facilitates Cisplatin (DDP) resistance and inhibits ferroptosis in endometrial cancer cells (131, 132). Additionally, LCN2 may regulate chemosensitivity to gemcitabine treatment in pancreatic cancer. However, further research is needed to explore the role of LCN2 in gemcitabine resistance (133). Overall, LCN2 is a key mediator of chemotherapy resistance across various cancers, warranting further research into its mechanisms and potential as a therapeutic target. However, lack of understanding of its specific mechanisms and effects in BCBM necessitates further comprehensive research to elucidate the role of LCN2 in this context.

## 7 LCN2 as a potential marker for diagnosis and therapy of BCBM

Based on the aforementioned findings, LCN2 has been identified as a facilitator of breast tumor invasion and metastasis by a variety of mechanisms: 1) induction of EMT, 2) enhancement of angiogenesis and lymphangiogenesis, 3) binding to MMP-9, 4) interaction with innate immune system, 5) disruption of BBB 6) modulation of brain microenvironment. These activities position LCN2 as a potential biomarker and therapeutic target for the prevention of brain metastasis in breast cancer.

In a murine model, elevation of LCN2 levels in the blood preceded the detection of brain metastasis by MRI (14). Notably, nearly all mice with markedly elevated LCN2 levels developed brain metastases, reinforcing the idea that elevated LCN2 can serve as an indicator of brain metastasis (14). In human studies, Lcn2 levels in urine and tissue samples from breast cancer patients have correlated with the presence of cancer status and poor prognosis (136).Moreover, the MMP-9/NGAL complex was detected in 86.36% of urine samples from breast cancer patients, contrasting with its absence in samples from healthy age and sex-matched controls (35). In a study of 55 female breast cancer patients revealed a significant correlation between LCN2 and MM9 levels, suggesting its potential utility in predicting MMP9 levels (63).

Therapies capable of reducing LCN2 secretion could offer significant benefits for patients with breast cancer that has metastasized to the brain. Mice deficient in LCN2 or treated with an LCN2 inhibitory monoclonal antibody shown a reduction in tumor growth and metastasis, attributed to the destabilization of the LCN2/MMP-9 complex, underscoring the potential of such interventions in addressing breast cancer brain metastasis (37). Targeting LCN2 with a specific monoclonal antibody has also been shown to attenuate the release of pro-inflammatory mediator and to reduce the permeability of BBB further indicating the effectiveness of this approach in curtailing brain metastasis (71).

A combined strategy involving the design of drug delivery systems that can silence LCN2 while simultaneously blocking other signaling pathways may offer a more effective way to impede brain metastasis by targeting multiple migratory routes. For instance, an Anti-CXCR4- targeted liposome encapsulating LCN2 siRNA effectively targets metastatic breast cancer cells and significantly blocks migration in triple-negative human breast cancer cells (88% for MDA-MB-436 and 92% for MDA-MB-231) along the CXCR4-CXCL12 axis (137). Efficient silencing of LCN2 by ICAM-1-targeted liposomes encapsulating LCN2 siRNA (ICAM-LCN2-LP) resulted in a remarkable decrease in vascular endothelial growth factor (VEGF) production and substantially suppressed angiogenesis in MDA-MB-231 cells that can metastasize to the brain, both *in vitro and in vivo* (136).

## 8 Conclusion and prospect

The field of targeted therapies is rapidly advancing, with LCN2 rising as an innovative therapeutic target. Researches indicated that LCN2 exerts a crucial influence on the progression of breast cancer brain metastasis through diverse mechanisms, including enhancement of proliferation and EMT, ECM remodeling and intravasation, disruption of the blood-brain barrier, modulation of the neuroinflammation and immune suppression in brain microenvironment. The multifaceted interactions of LCN2 with various cellular elements and signaling pathways position it as a central mediator in the metastatic cascade. The presence of elevated systemic levels of LCN2 is associated with a poor prognosis and enhanced metastatic capacity, highlighting its potential as a prognostic biomarker. Interventions such as targeting LCN2 with specific monoclonal antibodies or siRNA delivery systems have shown promise in curbing tumor growth and metastasis.

Despite these advances, significant knowledge gaps persist regarding LCN2. A key focus is investigating how LCN2 regulates the expression of epithelial and mesenchymal markers in CTCs. This could yield insights into their metastatic potential and provide a non-invasive method of predicting responses to therapy. Another critical area of research is the impact of the brain microenvironment on BrM; specifically, the influence of LCN2 on neurons and other cells such as microglia, oligodendrocytes, which warrants further investigation. Of utmost importance, future research should concentrate on devising effective therapeutic strategies aimed at inhibiting LCN2 activity, with the goal of enhancing the treatment and outcomes for patients afflicted with breast cancer brain metastasis.

## References

[1] Wilkinson L Gathani T. Understanding breast cancer as a global health concern. Br J Radiol. (2022) 95:20211033. doi: 10.1259/bjr.20211033 34905391 PMC8822551

[2] Bryan S Witzel I Borgmann K Oliveira-Ferrer L. Molecular mechanisms associated with brain metastases in HER2-positive and triple negative breast cancers. Cancers (Basel). (2021) 13(16):4137. doi: 10.3390/cancers13164137 34439289 PMC8392331

[3] Sperduto PW Kased N Roberge D Chao ST Shanley R Luo X. The effect of tumor subtype on the time from primary diagnosis to development of brain metastases and survival in patients with breast cancer. J Neurooncol. (2013) 112:467–72. doi: 10.1007/s11060-013-1083-9 23462853

[4] Kennecke H Yerushalmi R Woods R Cheang MC Voduc D Speers CH. Metastatic behavior of breast cancer subtypes. J Clin Oncol. (2010) 28:3271–7. doi: 10.1200/JCO.2009.25.9820 20498394

[5] Ivanova M Porta FM Giugliano F Frascarelli C Sajjadi E Venetis K. Breast cancer with brain metastasis: Molecular insights and clinical management. Genes. (2023) 14:1160. doi: 10.3390/genes14061160 37372340 PMC10298724

[6] Watase C Shiino S Shimoi T Noguchi E Kaneda T Yamamoto Y. Breast cancer brain metastasis-overview of disease state, treatment options and future perspectives. Cancers (Basel). (2021) 13(5):1078. doi: 10.3390/cancers13051078 33802424 PMC7959316

[7] Wrobel JK Toborek M. Blood-brain barrier remodeling during brain metastasis formation. Mol Med. (2016) 22:32–40. doi: 10.2119/molmed.2015.00207 26837070 PMC5004726

[8] Bonni S Brindley DN Chamberlain MD Daneshvar-Baghbadorani N Freywald A Hemmings DG. Breast tumor metastasis and its microenvironment: it takes both seed and soil to grow a tumor and target it for treatment. Cancers. (2024) 16:911. doi: 10.3390/cancers16050911 38473273 PMC10931297

[9] Liu Q Zhang H Jiang X Qian C Liu Z Luo D. Factors involved in cancer metastasis: a better understanding to "seed and soil" hypothesis. Mol Cancer. (2017) 16:176. doi: 10.1186/s12943-017-0742-4 29197379 PMC5712107

[10] Sun H Xu J Dai S Ma Y Sun T. Breast cancer brain metastasis: Current evidence and future directions. Cancer Med. (2023) 12:1007–24. doi: 10.1002/cam4.5021 PMC9883555 35822637

[11] Kobus T Zervantonakis IK Zhang Y McDannold NJ. Growth inhibition in a brain metastasis model by antibody delivery using focused ultrasound-mediated blood-brain barrier disruption. J Controlled Release. (2016) 238:281–8. doi: 10.1016/j.jconrel.2016.08.001 PMC5014601 27496633

[12] Barakeh DH Alsolme E Alqubaishi F Almutairi A Alhabeeb L Al Abdulmohsen S. Clinicopathologic and genomic characterizations of brain metastases using a comprehensive genomic panel. Front Med (Lausanne). (2022) 9:947456. doi: 10.3389/fmed.2022.947456 36507516 PMC9729258

[13] Dagogo-Jack I Shaw AT. Tumour heterogeneity and resistance to cancer therapies. Nat Rev Clin Oncol. (2018) 15:81–94. doi: 10.1038/nrclinonc.2017.166 29115304

[14] Adler O Zait Y Cohen N Blazquez R Doron H Monteran L. Reciprocal interactions between innate immune cells and astrocytes facilitate neuroinflammation and brain metastasis via lipocalin-2. Nat Cancer. (2023) 4:401–18. doi: 10.1038/s43018-023-00519-w 36797502

[15] Flower DR. The lipocalin protein family: A role in cell regulation. FEBS Lett. (1994) 354:7–11. doi: 10.1016/0014-5793(94)01078-1 7957904

[16] Ferreira AC Dá Mesquita S Sousa JC Correia-Neves M Sousa N Palha JA. From the periphery to the brain: Lipocalin-2, a friend or foe? Prog Neurobiol. (2015) 131:120–36. doi: 10.1016/j.pneurobio.2015.06.005 26159707

[17] Lim D Jeong JH Song J. Lipocalin 2 regulates iron homeostasis, neuroinflammation, and insulin resistance in the brains of patients with dementia: Evidence from the current literature. CNS Neurosci Ther. (2021) 27:883–94. doi: 10.1111/cns.13653 PMC8265939 33945675

[18] Kjeldsen L Johnsen AH Sengeløv H Borregaard N. Isolation and primary structure of NGAL, a novel protein associated with human neutrophil gelatinase. J Biol Chem. (1993) 268:10425–32. doi: 10.1016/S0021-9258(18)82217-7 7683678

[19] Jaberi SA Cohen A D’Souza C Abdulrazzaq YM Ojha S Bastaki S. Lipocalin-2: Structure, function, distribution and role in metabolic disorders. Biomedicine Pharmacotherapy. (2021) 142:112002. doi: 10.1016/j.biopha.2021.112002 34463264

[20] Goetz DH Willie ST Armen RS Bratt T Borregaard N Strong RK. Ligand preference inferred from the structure of neutrophil gelatinase associated lipocalin. Biochemistry. (2000) 39:1935–41. doi: 10.1021/bi992215v 10684642

[21] Hvidberg V Jacobsen C Strong RK Cowland JB Moestrup SK Borregaard N. The endocytic receptor megalin binds the iron transporting neutrophil-gelatinase-associated lipocalin with high affinity and mediates its cellular uptake. FEBS Lett. (2005) 579:773–7. doi: 10.1016/j.febslet.2004.12.031 15670845

[22] Devireddy LR Gazin C Zhu X Green MR. A cell-surface receptor for lipocalin 24p3 selectively mediates apoptosis and iron uptake. Cell. (2005) 123:1293–305. doi: 10.1016/j.cell.2005.10.027 16377569

[23] Xing C Wang X Cheng C Montaner J Mandeville E Leung W. Neuronal production of lipocalin-2 as a help-me signal for glial activation. Stroke. (2014) 45:2085–92. doi: 10.1161/STROKEAHA.114.005733 PMC4122238 24916903

[24] Asaf S Maqsood F Jalil J Sarfraz Z Sarfraz A Mustafa S. Lipocalin 2-not only a biomarker: a study of current literature and systematic findings of ongoing clinical trials. Immunol Res. (2023) 71:287–313. doi: 10.1007/s12026-022-09352-2 36529828 PMC9760530

[25] Bao G Clifton M Hoette TM Mori K Deng SX Qiu A. Iron traffics in circulation bound to a siderocalin (Ngal)-catechol complex. Nat Chem Biol. (2010) 6:602–9. doi: 10.1038/nchembio.402 PMC2907470 20581821

[26] Flo TH Smith KD Sato S Rodriguez DJ Holmes MA Strong RK. Lipocalin 2 mediates an innate immune response to bacterial infection by sequestrating iron. Nature. (2004) 432:917–21. doi: 10.1038/nature03104 15531878

[27] Srinivasan G Aitken JD Zhang B Carvalho FA Chassaing B Shashidharamurthy R. Lipocalin 2 deficiency dysregulates iron homeostasis and exacerbates endotoxin-induced sepsis. J Immunol. (2012) 189:1911–9. doi: 10.4049/jimmunol.1200892 PMC3411903 22786765

[28] Zhang J Wu Y Zhang Y LeRoith D Bernlohr DA Chen X. The role of lipocalin 2 in the regulation of inflammation in adipocytes and macrophages. Mol Endocrinol. (2008) 22:1416–26. doi: 10.1210/me.2007-0420 PMC2422824 18292240

[29] Roudkenar MH Halabian R Bahmani P Roushandeh AM Kuwahara Y Fukumoto M. Neutrophil gelatinase-associated lipocalin: A new antioxidant that exerts its cytoprotective effect independent on Heme Oxygenase-1. Free Radical Res. (2011) 45:810–9. doi: 10.3109/10715762.2011.581279 21545264

[30] Roudkenar MH Halabian R Ghasemipour Z Roushandeh AM Rouhbakhsh M Nekogoftar M. Neutrophil gelatinase-associated lipocalin acts as a protective factor against H2O2 toxicity. Arch Med Res. (2008) 39:560–6. doi: 10.1016/j.arcmed.2008.05.003 18662586

[31] Chiang K-C Yeh T-S Wu R-C Pang J-HS Cheng C-T Wang S-Y. Lipocalin 2 (LCN2) is a promising target for cholangiocarcinoma treatment and bile LCN2 level is a potential cholangiocarcinoma diagnostic marker. Sci Rep. (2016) 6:36138. doi: 10.1038/srep36138 27782193 PMC5080596

[32] Chaudhary N Choudhary BS Shah SG Khapare N Dwivedi N Gaikwad A. Lipocalin 2 expression promotes tumor progression and therapy resistance by inhibiting ferroptosis in colorectal cancer. Int J Cancer. (2021) 149:1495–511. doi: 10.1002/ijc.33711 34146401

[33] Godinho-Pereira J Vaz D Figueira I Aniceto-Romão J Krizbai I Malhó R. Breast cancer brain metastases: Implementation and characterization of a mouse model relying on Malignant cells inoculation in the carotid artery. Cells. (2023) 12(16):2076. doi: 10.3390/cells12162076 37626886 PMC10453310

[34] Santiago-Sánchez GS Pita-Grisanti V Quiñones-Díaz B Gumpper K Cruz-Monserrate Z Vivas-Mejía PE. Biological functions and therapeutic potential of lipocalin 2 in cancer. Int J Mol Sci. (2020) 21(12):4365. doi: 10.3390/ijms21124365 32575507 PMC7352275

[35] Fernández CA Yan L Louis G Yang J Kutok JL Moses MA. The matrix metalloproteinase-9/neutrophil gelatinase-associated lipocalin complex plays a role in breast tumor growth and is present in the urine of breast cancer patients. Clin Cancer Res. (2005) 11:5390–5. doi: 10.1158/1078-0432.CCR-04-2391 16061852

[36] Li S-H Hawthorne VS Neal CL Sanghera S Xu J Yang J. Upregulation of neutrophil gelatinase–associated lipocalin by erbB2 through nuclear factor-κB activation. Cancer Res. (2009) 69:9163–8. doi: 10.1158/0008-5472.CAN-09-2483 PMC2794902 19951994

[37] Leng X Ding T Lin H Wang Y Hu L Hu J. Inhibition of lipocalin 2 impairs breast tumorigenesis and metastasis. Cancer Res. (2009) 69:8579–84. doi: 10.1158/0008-5472.CAN-09-1934 19887608

[38] Villodre ES Hu X Larson R Finetti P Gomez K Balema W. Lipocalin 2 promotes inflammatory breast cancer tumorigenesis and skin invasion. Mol Oncol. (2021) 15:2752–65. doi: 10.1002/1878-0261.13074 PMC8486564 34342930

[39] Tan M He Y Yi J Chen J Guo Q Liao N. WTAP mediates NUPR1 regulation of LCN2 through m(6)A modification to influence ferroptosis, thereby promoting breast cancer proliferation, migration and invasion. Biochem Genet. (2024) 62:876–91. doi: 10.1007/s10528-023-10423-8 37477758

[40] Paduch R. The role of lymphangiogenesis and angiogenesis in tumor metastasis. Cell Oncol (Dordr). (2016) 39:397–410. doi: 10.1007/s13402-016-0281-9 27126599 PMC5052283

[41] Zhang L Wang H. FTY720 in CNS injuries: Molecular mechanisms and therapeutic potential. Brain Res Bull. (2020) 164:75–82. doi: 10.1016/j.brainresbull.2020.08.013 32846199

[42] Yang J McNeish B Butterfield C Moses MA. Lipocalin 2 is a novel regulator of angiogenesis in human breast cancer. FASEB J. (2013) 27:45–50. doi: 10.1096/fj.12-211730 22982376 PMC3528324

[43] Schoppmann SF Birner P Stöckl J Kalt R Ullrich R Caucig C. Tumor-associated macrophages express lymphatic endothelial growth factors and are related to peritumoral lymphangiogenesis. Am J Pathol. (2002) 161:947–56. doi: 10.1016/S0002-9440(10)64255-1 PMC1867252 12213723

[44] Ji RC. Macrophages are important mediators of either tumor- or inflammation-induced lymphangiogenesis. Cell Mol Life Sci. (2012) 69:897–914. doi: 10.1007/s00018-011-0848-6 21984600 PMC11114502

[45] Jung M Ören B Mora J Mertens C Dziumbla S Popp R. Lipocalin 2 from macrophages stimulated by tumor cell–derived sphingosine 1-phosphate promotes lymphangiogenesis and tumor metastasis. Sci Signaling. (2016) 9:ra64–4. doi: 10.1126/scisignal.aaf3241 27353364

[46] Bakir B Chiarella AM Pitarresi JR Rustgi AK EMT MET. Plasticity, and tumor metastasis. Trends Cell Biol. (2020) 30:764–76. doi: 10.1016/j.tcb.2020.07.003 PMC7647095 32800658

[47] Yang J Bielenberg DR Rodig SJ Doiron R Clifton MC Kung AL. Lipocalin 2 promotes breast cancer progression. Proc Natl Acad Sci. (2009) 106:3913–8. doi: 10.1073/pnas.0810617106 PMC2656179 19237579

[48] Chiang KC Hsu SY Lin SJ Yeh CN Pang JH Wang SY. PTEN insufficiency increases breast cancer cell metastasis in vitro and *in vivo* in a xenograft zebrafish model. Anticancer Res. (2016) 36:3997–4005. 27466505

[49] Guo P Yang J Huang J Auguste DT Moses MA. Therapeutic genome editing of triple-negative breast tumors using a noncationic and deformable nanolipogel. Proc Natl Acad Sci. (2019) 116:18295–303. doi: 10.1073/pnas.1904697116 PMC6744870 31451668

[50] Chiang K-C Yeh T-S Chen S-C Pang J-HS Yeh C-N Hsu J-T. The vitamin D analog, MART-10, attenuates triple negative breast cancer cells metastatic potential. Int J Mol Sci. (2016) 17:606. doi: 10.3390/ijms17040606 27110769 PMC4849057

[51] Seth P Porter D Lahti-Domenici J Geng Y Richardson A Polyak K. Cellular and molecular targets of estrogen in normal human breast tissue1. Cancer Res. (2002) 62:4540–4. 12183401

[52] Tyagi A Sharma S Wu K Wu S-Y Xing F Liu Y. Nicotine promotes breast cancer metastasis by stimulating N2 neutrophils and generating pre-metastatic niche in lung. Nat Commun. (2021) 12:474. doi: 10.1038/s41467-020-20733-9 33473115 PMC7817836

[53] Leng X Wu Y Arlinghaus RB. Relationships of lipocalin 2 with breast tumorigenesis and metastasis. J Cell Physiol. (2011) 226:309–14. doi: 10.1002/jcp.22403 20857428

[54] Xu WX Zhang J Hua YT Yang SJ Wang DD Tang JH. An integrative pan-cancer analysis revealing LCN2 as an oncogenic immune protein in tumor microenvironment. Front Oncol. (2020) 10:605097. doi: 10.3389/fonc.2020.605097 33425761 PMC7786136

[55] Ören B Urosevic J Mertens C Mora J Guiu M Gomis RR. Tumour stroma-derived lipocalin-2 promotes breast cancer metastasis. J Pathol. (2016) 239:274–85. doi: 10.1002/path.4724 27038000

[56] Malone MK Smrekar K Park S Blakely B Walter A Nasta N. Cytokines secreted by stromal cells in TNBC microenvironment as potential targets for cancer therapy. Cancer Biol Ther. (2020) 21:560–9. doi: 10.1080/15384047.2020.1739484 PMC7515526 32213106

[57] Crescenzi E Leonardi A Pacifico F. NGAL as a potential target in tumor microenvironment. Int J Mol Sci. (2021) 22(22):12333. doi: 10.3390/ijms222212333 34830212 PMC8623964

[58] Yuan Z Li Y Zhang S Wang X Dou H Yu X. Extracellular matrix remodeling in tumor progression and immune escape: from mechanisms to treatments. Mol Cancer. (2023) 22:48. doi: 10.1186/s12943-023-01744-8 36906534 PMC10007858

[59] Nazir SU Kumar R Singh A Khan A Tanwar P Tripathi R. Breast cancer invasion and progression by MMP-9 through Ets-1 transcription factor. Gene. (2019) 711:143952. doi: 10.1016/j.gene.2019.143952 31265880

[60] Owyong M Chou J van den Bijgaart RJ Kong N Efe G Maynard C. MMP9 modulates the metastatic cascade and immune landscape for breast cancer anti-metastatic therapy. Life Sci Alliance. (2019) 2(6):e201800226. doi: 10.26508/lsa.201800226 31727800 PMC6856766

[61] Yan L Borregaard N Kjeldsen L Moses MA. The high molecular weight urinary matrix metalloproteinase (MMP) activity is a complex of gelatinase B/MMP-9 and neutrophil gelatinase-associated lipocalin (NGAL). Modulation of MMP-9 activity by NGAL. J Biol Chem. (2001) 276:37258–65. doi: 10.1074/jbc.M106089200 11486009

[62] Wang L Li H Wang J Gao W Lin Y Jin W. C/EBP ζ targets to neutrophil gelatinase-associated lipocalin (NGAL) as a repressor for metastasis of MDA-MB-231 cells. Biochim Biophys Acta (BBA) - Mol Cell Res. (2011) 1813:1803–13. doi: 10.1016/j.bbamcr.2011.06.010 21741997

[63] Bahrun U Wildana W Rahmawati H Kurniawan LB Hamdani W. Lipocalin 2 could predict circulating MMP9 levels in patients with breast cancer. Breast Dis. (2021) 40:S115–s117. doi: 10.3233/BD-219017 34057126

[64] Cheng G Sun X Wang J Xiao G Wang X Fan X. HIC1 silencing in triple-negative breast cancer drives progression through misregulation of LCN2. Cancer Res. (2014) 74:862–72. doi: 10.1158/0008-5472.CAN-13-2420 24295734

[65] Lozar T Gersak K Cemazar M Kuhar CG Jesenko T. The biology and clinical potential of circulating tumor cells. Radiol Oncol. (2019) 53:131–47. doi: 10.2478/raon-2019-0024 PMC6572494 31104002

[66] Corti C Antonarelli G Criscitiello C Lin NU Carey LA Cortés J. Targeting brain metastases in breast cancer. Cancer Treat Rev. (2022) 103:102324. doi: 10.1016/j.ctrv.2021.102324 34953200

[67] Arvanitis CD Ferraro GB Jain RK. The blood–brain barrier and blood–tumour barrier in brain tumours and metastases. Nat Rev Cancer. (2020) 20:26–41. doi: 10.1038/s41568-019-0205-x 31601988 PMC8246629

[68] Manu DR Slevin M Barcutean L Forro T Boghitoiu T Balasa R. Astrocyte involvement in blood-brain barrier function: A critical update highlighting novel, complex, neurovascular interactions. Int J Mol Sci. (2023) 24(24):17146. doi: 10.3390/ijms242417146 38138976 PMC10743219

[69] Benz F Liebner S. Structure and function of the blood-brain barrier (BBB). Handb Exp Pharmacol. (2022) 273:3–31. doi: 10.1007/164_2020_404 33249527

[70] Burek M König A Lang M Fiedler J Oerter S Roewer N. Hypoxia-induced microRNA-212/132 alter blood-brain barrier integrity through inhibition of tight junction-associated proteins in human and mouse brain microvascular endothelial cells. Transl Stroke Res. (2019) 10:672–83. doi: 10.1007/s12975-018-0683-2 PMC6842347 30617994

[71] Wang G Weng Y-C Chiang I-C Huang Y-T Liao Y-C Chen Y-C. Neutralization of lipocalin-2 diminishes stroke-reperfusion injury. Int J Mol Sci. (2020) 21:6253. doi: 10.3390/ijms21176253 32872405 PMC7503651

[72] Egashira Y Hua Y Keep RF Iwama T Xi G. Lipocalin 2 and blood-brain barrier disruption in white matter after experimental subarachnoid hemorrhage. Acta Neurochir Suppl. (2016) 121:131–4. doi: 10.1007/978-3-319-18497-5_23 26463936

[73] Jin M Kim J-H Jang E Lee YM Han HS Woo DK. Lipocalin-2 deficiency attenuates neuroinflammation and brain injury after transient middle cerebral artery occlusion in mice. J Cereb Blood Flow Metab. (2014) 34:1306–14. doi: 10.1038/jcbfm.2014.83 PMC4126090 24780901

[74] Yang J Ran M Li H Lin Y Ma K Yang Y. New insight into neurological degeneration: Inflammatory cytokines and blood-brain barrier. Front Mol Neurosci. (2022) 15:1013933. doi: 10.3389/fnmol.2022.1013933 36353359 PMC9637688

[75] Mondal A Bose D Saha P Sarkar S Seth R Kimono D. Lipocalin 2 induces neuroinflammation and blood-brain barrier dysfunction through liver-brain axis in murine model of nonalcoholic steatohepatitis. J Neuroinflamm. (2020) 17:201. doi: 10.1186/s12974-020-01876-4 PMC7335438 32622362

[76] Kadry H Noorani B Cucullo L. A blood–brain barrier overview on structure, function, impairment, and biomarkers of integrity. Fluids Barriers CNS. (2020) 17:69. doi: 10.1186/s12987-020-00230-3 33208141 PMC7672931

[77] Kim JH Ko PW Lee HW Jeong JY Lee MG Kim JH. Astrocyte-derived lipocalin-2 mediates hippocampal damage and cognitive deficits in experimental models of vascular dementia. Glia. (2017) 65:1471–90. doi: 10.1002/glia.23174 28581123

[78] Argaw AT Asp L Zhang J Navrazhina K Pham T Mariani JN. Astrocyte-derived VEGF-A drives blood-brain barrier disruption in CNS inflammatory disease. J Clin Invest. (2012) 122:2454–68. doi: 10.1172/JCI60842 PMC3386814 22653056

[79] Argaw AT Gurfein BT Zhang Y Zameer A John GR. VEGF-mediated disruption of endothelial CLN-5 promotes blood-brain barrier breakdown. Proc Natl Acad Sci. (2009) 106:1977–82. doi: 10.1073/pnas.0808698106 PMC2644149 19174516

[80] Qiu YM Zhang CL Chen AQ Wang HL Zhou YF Li YN. Immune cells in the BBB disruption after acute ischemic stroke: targets for immune therapy? Front Immunol. (2021) 12:678744. doi: 10.3389/fimmu.2021.678744 34248961 PMC8260997

[81] Opdenakker G Van den Steen PE Dubois B Nelissen I Van Coillie E Masure S. Gelatinase B functions as regulator and effector in leukocyte biology. J Leukoc Biol. (2001) 69:851–9. doi: 10.1189/jlb.69.6.851 11404367

[82] Obermeier B Daneman R Ransohoff RM. Development, maintenance and disruption of the blood-brain barrier. Nat Med. (2013) 19:1584–96. doi: 10.1038/nm.3407 PMC4080800 24309662

[83] Kang R Gamdzyk M Lenahan C Tang J Tan S Zhang JH. The dual role of microglia in blood-brain barrier dysfunction after stroke. Curr Neuropharmacol. (2020) 18:1237–49. doi: 10.2174/1570159X18666200529150907 PMC7770642 32469699

[84] Cheng L Xing H Mao X Li L Li X Li Q. Lipocalin-2 promotes m1 macrophages polarization in a mouse cardiac ischaemia-reperfusion injury model. Scand J Immunol. (2015) 81:31–8. doi: 10.1111/sji.12245 25359467

[85] Ronaldson PT Davis TP. Regulation of blood-brain barrier integrity by microglia in health and disease: A therapeutic opportunity. J Cereb Blood Flow Metab. (2020) 40:S6–s24. doi: 10.1177/0271678X20951995 32928017 PMC7687032

[86] Thompson BJ Ronaldson PT. Drug delivery to the ischemic brain. Adv Pharmacol. (2014) 71:165–202. doi: 10.1016/bs.apha.2014.06.013 25307217 PMC4281266

[87] Chen AQ Fang Z Chen XL Yang S Zhou YF Mao L. Microglia-derived TNF-α mediates endothelial necroptosis aggravating blood brain-barrier disruption after ischemic stroke. Cell Death Dis. (2019) 10:487. doi: 10.1038/s41419-019-1716-9 31221990 PMC6586814

[88] Aslam M Ahmad N Srivastava R Hemmer B. TNF-alpha induced NFκB signaling and p65 (RelA) overexpression repress Cldn5 promoter in mouse brain endothelial cells. Cytokine. (2012) 57:269–75. doi: 10.1016/j.cyto.2011.10.016 22138107

[89] Ni Y Teng T Li R Simonyi A Sun GY Lee JC. TNFα alters occludin and cerebral endothelial permeability: Role of p38MAPK. PLoS One. (2017) 12:e0170346. doi: 10.1371/journal.pone.0170346 28170408 PMC5295672

[90] Ding XW Sun X Shen XF Lu Y Wang JQ Sun ZR. Propofol attenuates TNF-α-induced MMP-9 expression in human cerebral microvascular endothelial cells by inhibiting Ca(2+)/CAMK II/ERK/NF-κB signaling pathway. Acta Pharmacol Sin. (2019) 40:1303–13. doi: 10.1038/s41401-019-0258-0 PMC6786358 31235816

[91] Boire A Brastianos PK Garzia L Valiente M. Brain metastasis. Nat Rev Cancer. (2020) 20:4–11. doi: 10.1038/s41568-019-0220-y 31780784

[92] Klotz R Thomas A Teng T Han SM Iriondo O Li L. Circulating tumor cells exhibit metastatic tropism and reveal brain metastasis drivers. Cancer Discovery. (2020) 10:86–103. doi: 10.1158/2159-8290.CD-19-0384 31601552 PMC6954305

[93] Gonzalez H Mei W Robles I Hagerling C Allen BM Hauge Okholm TL. Cellular architecture of human brain metastases. Cell. (2022) 185:729–745.e20. doi: 10.1016/j.cell.2021.12.043 35063085 PMC8857062

[94] Zou Y Ye F Kong Y Hu X Deng X Xie J. The single-cell landscape of intratumoral heterogeneity and the immunosuppressive microenvironment in liver and brain metastases of breast cancer. Adv Sci (Weinh). (2023) 10:e2203699. doi: 10.1002/advs.202203699 36529697 PMC9929130

[95] Chung B Esmaeili AA Gopalakrishna-Pillai S Murad JP Andersen ES Kumar Reddy N. Human brain metastatic stroma attracts breast cancer cells via chemokines CXCL16 and CXCL12. NPJ Breast Cancer. (2017) 3:6. doi: 10.1038/s41523-017-0008-8 28649646 PMC5460196

[96] Dankner M Maritan SM Priego N Nadaf J Nkili A Zhuang R. Abstract 1569: pSTAT3+ stromal cells drive the invasive growth of brain metastases. Cancer Res. (2022) 82:1569–9. doi: 10.1158/1538-7445.AM2022-1569

[97] Feng Y Hu X Zhang Y Wang Y. The role of microglia in brain metastases: Mechanisms and strategies. Aging Dis. (2024) 15:169–85. doi: 10.14336/AD.2023.0514 PMC10796095 37307835

[98] Lee S Park JY Lee WH Kim H Park HC Mori K. Lipocalin-2 is an autocrine mediator of reactive astrocytosis. J Neurosci. (2009) 29:234–49. doi: 10.1523/JNEUROSCI.5273-08.2009 PMC6664907 19129400

[99] Lee S Lee J Kim S Park JY Lee WH Mori K. A dual role of lipocalin 2 in the apoptosis and deramification of activated microglia. J Immunol. (2007) 179:3231–41. doi: 10.4049/jimmunol.179.5.3231 17709539

[100] Li J Xu P Hong Y Xie Y Peng M Sun R. Lipocalin-2-mediated astrocyte pyroptosis promotes neuroinflammatory injury via NLRP3 inflammasome activation in cerebral ischemia/reperfusion injury. J Neuroinflamm. (2023) 20:148. doi: 10.1186/s12974-023-02819-5 PMC10288712 37353794

[101] Nam Y Kim JH Seo M Kim JH Jin M Jeon S. Lipocalin-2 protein deficiency ameliorates experimental autoimmune encephalomyelitis: the pathogenic role of lipocalin-2 in the central nervous system and peripheral lymphoid tissues. J Biol Chem. (2014) 289:16773–89. doi: 10.1074/jbc.M113.542282 PMC4059121 24808182

[102] Zhao R-Y Wei PJ Sun X Zhang DH He QY Liu J. Role of lipocalin 2 in stroke. Neurobiol Dis. (2023) 179:106044. doi: 10.1016/j.nbd.2023.106044 36804285

[103] Jang E Kim J-H Lee S Kim J-H Seo J-W Jin M. Phenotypic polarization of activated astrocytes: the critical role of lipocalin-2 in the classical inflammatory activation of astrocytes. J Immunol. (2013) 191:5204–19. doi: 10.4049/jimmunol.1301637 24089194

[104] McFarland BC Benveniste EN. Reactive astrocytes foster brain metastases via STAT3 signaling. Ann Transl Med. (2019) 7:S83. doi: 10.21037/atm 31576292 PMC6685879

[105] Afridi R Kim J-H Bhusal A Lee W-H Suk K. Lipocalin-2 as a mediator of neuroimmune communication. J Leukocyte Biol. (2023) 116:357–368. doi: 10.1093/jleuko/qiad157 38149462

[106] Sacco A Battaglia AM Botta C Aversa I Mancuso S Costanzo F. Iron metabolism in the tumor microenvironment-implications for anti-cancer immune response. Cells. (2021) 10(2):303. doi: 10.3390/cells10020303 33540645 PMC7913036

[107] Mertens C Schnetz M Rehwald C Grein S Elwakeel E Weigert A. Iron-bound lipocalin-2 from tumor-associated macrophages drives breast cancer progression independent of ferroportin. Metabolites. (2021) 11:180. doi: 10.3390/metabo11030180 33808732 PMC8003561

[108] Krizanac M Mass Sanchez PB Weiskirchen R Schröder SK. Overview of the expression patterns and roles of Lipocalin 2 in the reproductive system. Front Endocrinol (Lausanne). (2024) 15:1365602. doi: 10.3389/fendo.2024.1365602 38645429 PMC11026566

[109] Jung M Weigert A Mertens C Rehwald C Brüne B. Iron handling in tumor-associated macrophages-is there a new role for lipocalin-2? Front Immunol. (2017) 8:1171. doi: 10.3389/fimmu.2017.01171 28979267 PMC5611490

[110] Mertens C Mora J Ören B Grein S Winslow S Scholich K. Macrophage-derived lipocalin-2 transports iron in the tumor microenvironment. Oncoimmunology. (2018) 7:e1408751. doi: 10.1080/2162402X.2017.1408751 29399416 PMC5790355

[111] Jung M Weigert A Tausendschön M Mora J Ören B Sola A. Interleukin-10-induced neutrophil gelatinase-associated lipocalin production in macrophages with consequences for tumor growth. Mol Cell Biol. (2012) 32:3938–48. doi: 10.1128/MCB.00413-12 PMC3457542 22851691

[112] Chi Y Remsik J Kiseliovas V Derderian C Sener U Alghader M. Cancer cells deploy lipocalin-2 to collect limiting iron in leptomeningeal metastasis. Science. (2020) 369:276–82. doi: 10.1126/science.aaz2193 PMC7816199 32675368

[113] Uchikawa E Chen Z Xiao G-Y Zhang X. Bai, X.-c., Structural basis of the activation of c-MET receptor. Nat Commun. (2021) 12:4074. doi: 10.1038/s41467-021-24367-3 34210960 PMC8249616

[114] Liu Y Smith MR Wang Y D'Agostino R Jr. Ruiz J Lycan T. c-met mediated cytokine network promotes brain metastasis of breast cancer by remodeling neutrophil activities. Cancers (Basel). (2023) 15(9):2626. doi: 10.3390/cancers15092626 37174093 PMC10177081

[115] Miserocchi G Bocchini M Cortesi M Arienti C De Vita A Liverani C. Combining preclinical tools and models to unravel tumor complexity: Jump into the next dimension. Front Immunol. (2023) 14:1171141. doi: 10.3389/fimmu.2023.1171141 37033986 PMC10080004

[116] Durinikova E Buzo K Arena S. Preclinical models as patients' avatars for precision medicine in colorectal cancer: past and future challenges. J Exp Clin Cancer Res. (2021) 40:185. doi: 10.1186/s13046-021-01981-z 34090508 PMC8178911

[117] Neal JT Li X Zhu J Giangarra V Grzeskowiak CL Ju J. Organoid modeling of the tumor immune microenvironment. Cell. (2018) 175:1972–1988.e16. doi: 10.1016/j.cell.2018.11.021 30550791 PMC6656687

[118] Nath S Devi GR. Three-dimensional culture systems in cancer research: Focus on tumor spheroid model. Pharmacol Ther. (2016) 163:94–108. doi: 10.1016/j.pharmthera.2016.03.013 27063403 PMC4961208

[119] Clevers H. Modeling development and disease with organoids. Cell. (2016) 165:1586–97. doi: 10.1016/j.cell.2016.05.082 27315476

[120] Imparato G Urciuolo F Netti PA. Organ on chip technology to model cancer growth and metastasis. Bioengineering (Basel). (2022) 9(1):28. doi: 10.3390/bioengineering9010028 35049737 PMC8772984

[121] Zhang I Lépine P Han C Lacalle-Aurioles M Chen CX-Q Haag R. Nanotherapeutic modulation of human neural cells and glioblastoma in organoids and monocultures. Cells. (2020) 9:2434. doi: 10.3390/cells9112434 33171886 PMC7695149

[122] Shen P Jia Y Zhou W Zheng W Wu Y Qu S. A biomimetic liver cancer on-a-chip reveals a critical role of LIPOCALIN-2 in promoting hepatocellular carcinoma progression. Acta Pharm Sin B. (2023) 13:4621–37. doi: 10.1016/j.apsb.2023.04.010 PMC10638501 37969730

[123] Baskar R Lee KA Yeo R Yeoh KW. Cancer and radiation therapy: current advances and future directions. Int J Med Sci. (2012) 9:193–9. doi: 10.7150/ijms.3635 PMC3298009 22408567

[124] Liu YP Zheng CC Huang YN He ML Xu WW Li B. Molecular mechanisms of chemo- and radiotherapy resistance and the potential implications for cancer treatment. MedComm. (2020) 2:315–40. doi: 10.1002/mco2.55 PMC8554658 34766149

[125] Shiiba M Saito K Fushimi K Ishigami T Shinozuka K Nakashima D. Lipocalin-2 is associated with radioresistance in oral cancer and lung cancer cells. Int J Oncol. (2013) 42:1197–204. doi: 10.3892/ijo.2013.1815 23403985

[126] Zhang MX Wang L Zeng L Tu ZW. Corrigendum: LCN2 is a potential biomarker for radioresistance and recurrence in nasopharyngeal carcinoma. Front Oncol. (2021) 11:670714. doi: 10.3389/fonc.2021.670714 33816321 PMC8016410

[127] Yu D-S Wu C-L Ping S-Y Huang Y-L Shen K-H. NGAL can alternately mediate sunitinib resistance in renal cell carcinoma. J Urol. (2014) 192:559–66. doi: 10.1016/j.juro.2013.12.049 24423438

[128] Zheng LT Lee S Yin GN Mori K Suk K. Down-regulation of lipocalin 2 contributes to chemoresistance in glioblastoma cells. J Neurochemistry. (2009) 111:1238–51. doi: 10.1111/j.1471-4159.2009.06410.x 19860839

[129] Monisha J Roy NK Padmavathi G Banik K Bordoloi D Khwairakpam AD. NGAL is downregulated in oral squamous cell carcinoma and leads to increased survival, proliferation, migration and chemoresistance. Cancers. (2018) 10:228. doi: 10.3390/cancers10070228 29996471 PMC6071146

[130] Kim I Kim KS Kwon OS Cha HJ Park KS. Ell3 stimulates 5-FU resistance in a breast cancer cell line. Oncol Lett. (2017) 13:4173–9. doi: 10.3892/ol.2017.5996 PMC5452875 28588704

[131] Shi C Wang C Fu Z Liu J Zhou Y Cheng B. Lipocalin 2 (LCN2) confers acquired resistance to almonertinib in NSCLC through LCN2-MMP-9 signaling pathway. Pharmacol Res. (2024) 201:107088. doi: 10.1016/j.phrs.2024.107088 38295916

[132] Jiang J Zhu J Qiu P Ni J Zhu W Wang X. HNRNPA2B1-mediated m6A modification of FOXM1 promotes drug resistance and inhibits ferroptosis in endometrial cancer via regulation of LCN2. Funct Integr Genomics. (2023) 24:3. doi: 10.1007/s10142-023-01279-7 38091112

[133] Zhang H Wu P Guo C Zhang C Zhao Y Tan D. Lipocalin 2 may be a key factor regulating the chemosensitivity of pancreatic cancer to gemcitabine. Biochem Biophysics Rep. (2022) 31:101291. doi: 10.1016/j.bbrep.2022.101291 PMC9166468 35669987

[134] Živalj M Van Ginderachter JA Stijlemans B. Lipocalin-2: A nurturer of tumor progression and a novel candidate for targeted cancer therapy. Cancers. (2023) 15:5159. doi: 10.3390/cancers15215159 37958332 PMC10648573

[135] Roudkenar MH Kuwahara Y Baba T Roushandeh AM Ebishima S Abe S. Oxidative stress induced lipocalin 2 gene expression: addressing its expression under the harmful conditions. J Radiat Res. (2007) 48:39–44. doi: 10.1269/jrr.06057 17229997

[136] Guo P Yang J Jia D Moses MA Auguste DT. ICAM-1-targeted, lcn2 siRNA-encapsulating liposomes are potent anti-angiogenic agents for triple negative breast cancer. Theranostics. (2016) 6:1–13. doi: 10.7150/thno.12167 26722369 PMC4679350

[137] Guo P You JO Yang J Jia D Moses MA Auguste DT. Inhibiting metastatic breast cancer cell migration via the synergy of targeted, pH-triggered siRNA delivery and chemokine axis blockade. Mol Pharm. (2014) 11:755–65. doi: 10.1021/mp4004699 PMC3993942 24467226

